# Characteristics and possible mechanisms of formation of microinversions distinguishing human and chimpanzee genomes

**DOI:** 10.1038/s41598-021-04621-w

**Published:** 2022-01-12

**Authors:** Nadezhda A. Potapova, Alexey S. Kondrashov, Sergei M. Mirkin

**Affiliations:** 1 Institute for Information Transmission Problems (Kharkevich Institute), Russian Academy of Sciences, Moscow, Russia 127051; 2 Department of Ecology and Evolutionary Biology, University of Michigan, Ann Arbor, MI 48109 USA; 3 Department of Biology, Tufts University, Medford, MA 02155 USA

**Keywords:** Genomic instability, Genetics, Genomics, Genome evolution

## Abstract

Genomic inversions come in various sizes. While long inversions are relatively easy to identify by aligning high-quality genome sequences, unambiguous identification of microinversions is more problematic. Here, using a set of extra stringent criteria to distinguish microinversions from other mutational events, we describe microinversions that occurred after the divergence of humans and chimpanzees. In total, we found 59 definite microinversions that range from 17 to 33 nucleotides in length. In majority of them, human genome sequences matched exactly the reverse-complemented chimpanzee genome sequences, implying that the inverted DNA segment was copied precisely. All these microinversions were flanked by perfect or nearly perfect inverted repeats pointing to their key role in their formation. Template switching at inverted repeats during DNA replication was previously discussed as a possible mechanism for the microinversion formation. However, many of definite microinversions found by us cannot be easily explained via template switching owing to the combination of the short length and imperfect nature of their flanking inverted repeats. We propose a novel, alternative mechanism that involves repair of a double-stranded break within the inverting segment via microhomology-mediated break-induced replication, which can consistently explain all definite microinversion events.

## Introduction

A pure inversion is a mutation that replaces a segment of the genome with its reverse complement sequence. Inversions are routinely found in studies of genetic variation both within species^1^ and between species^2^. Because the rate of inversions is much lower than that of single nucleotide substitutions and of short deletions and insertions^3,4^, they comprise only a small proportion of all genetic changes. Thus, to detect large number of inversions, it may be necessary to compare genomes of species that are separated from each other by a relatively long evolutionary path.

Inversions can vary in length dramatically^5–7^, which leads to different potential pitfalls that complicate ascertaining long and short inversions. Detection of long inversions can be hindered by incorrect genome assembly because most non-human genomes were sequenced via short-read platforms. Only comparison of high-quality genomes and usage of stringent parameters for genome assembly and alignment can produce reliable results. For example, 252 long inversions caused by retrotransposons were detected with high confidence on the human-chimpanzee path^8^.

Detection of short inversions (with lengths below 100 nucleotides), sometimes referred to as “ultra microinversions”^5^, “submicroscopic inversions”^7^ or “pico inversions”^6^, requires distinguishing them from products of other mechanisms that can also produce segments of low similarity (“bubbles”) in genome alignments, such as clustered small-scale mutations, mutational hotspots or simply a large number of independent small-scale mutations that occurred by chance within a short segment of the genome^2,9,10^. Moreover, multiple nucleotide substitutions, if they accumulated within a microinversion after it has occurred, can render it unrecognizable. Thus, identifying microinversions that occurred on a long evolutionary path may be practically impossible^2,11–13^.

Conversely, the more similar the genomes of two compared species are, the shorter the microinversions are that can be reliably detected. For example, human and chimpanzee genomes, which are > 98% identical if long deletions and insertions are ignored, and ~ 95% identical, if all genetic differences are considered^14^, provide an excellent system for ascertaining microinversions. Several comparative analyses were performed for these species, some of which reported thousands of microinversions^5,9,13,15^. Note, however, that the outcomes of these analyses differ significantly, likely due to different methods of filtering used in those studies.

Resolving the differences in the detection and analysis of microinversion is important for several reasons. First, it was hypothesized that microinversions may play a role in human genetic diseases and are overrepresented in cancer genomes^13,16^. Second, being such rare events, microinversions are very useful for phylogenetic inferences^2^ because the probability of homoplasy, due to repeated origins of the same microinversion, is extremely low. Third, they may help elucidate genetic relationships between human populations as sensitive markers for separating populations^17^.

Here we describe definite microinversion that occurred after the divergence of humans and chimpanzees, which were identified using a set of extra stringent criteria. Majority of them were pure inversions, i.e. human genome sequences matched exactly to the reverse-complemented chimpanzee sequences. All these microinversions were flanked by perfect or nearly perfect inverted repeats, strongly suggesting that they play a key role in the origin of microinversions. While template switching at inverted repeats was previously discussed as a possible mechanism for the microinversion formation^18–20^, many of definite microinversions detected by us cannot be easily explained by this mechanism. We, thus, propose a novel, alternative mechanism stipulating that microinversions emerge in course of double-strand break repair.

## Results

### Identifying microinversions between human-chimpanzee and chimpanzee-bonobo genome pairs

Ascertainment of microinversions by comparison of similar genomes appears to be a straightforward task because a microinversion produces a segment of the alignment where the sequence of one species is (nearly) identical to reverse-complemented sequence of the other species. Still, one needs to distinguish microinversions from other phenomena that can produce similar outcomes^9^.

As long as a tool that is used for comparing genomes is not specifically searching for microinversions, they present in the alignment as “bubbles”, or segments of low similarity. Long alignments routinely contain multiple bubbles, which are particularly conspicuous when the two compared genomes are generally very similar to each other. However, most of these bubbles are not products of microinversions. Thus, the key problem is to specifically recognize bubbles which resulted from microinversions.

How can a bubble emerge? A microinversion, an individual event of some other kind that involves many nucleotide sites, and a clump of many separate single-nucleotide substitutions^11^, and/or other small-scale mutations are the three options. Difficulty in discriminating between these options is the main reason why previous studies reported vastly different numbers of microinversions on the evolutionary path connecting humans and chimpanzees^5,6,21^.

How to distinguish between these three options? We believe that it is impossible to reliably recognize a microinversion that is too short. Indeed, a one nucleotide long microinversion cannot be distinguished from the corresponding transversion. It is a moot question, therefore, whether such microinversions exist. In general, a microinversion of a genome segment of length N looks like a succession of N single-nucleotide substitutions. Obviously, when N is small, even a bubble consisting of two genome segments of equal lengths such that the sequence of one segment coincides with the reverse-complemented sequences of the other segment (say, AG–CT, with N = 2) does not necessarily imply that a microinversion occurred. Several independent substitutions, or a complex mutation that produced such a pattern accidentally, cannot be ruled out. By contrast, a long enough microinversion (say, of N = 27, resulting in a bubble GCAGATCATCCTTTATTCCTACCTTGT—ACAAGGTAGGAATAAAGGATGATCTGC, see Table S1) is clearly recognizable because the probability of an accidental origin of such a bubble by any other means is vanishingly low.

What is the minimal length of a microinversion that can be reliably recognized as such by comparing the human and chimpanzee genomes? Because these two genomes differ from each other at ~ 1 nucleotide site out of 100, the probability that a bubble of length N appears as a result of independent single-nucleotide substitutions is a succession of N mismatches, is (1/100)^N^. Thus, a priori*,* in the alignment of length of ~ 1 billion nucleotides, this mechanism can generate bubbles of lengths only up to 5 nucleotides, and one might optimistically conclude that a microinversion of N > 5 can be reliably recognized.

However, this does not seem to be the case. Human-chimpanzee genome alignment contains 85,410 bubbles with lengths above 5 nucleotides, including 28,113 with lengths above 10 (Table S2). Clearly, a vast majority of these bubbles are not microinversions, since the sequence of one genome segment bears no similarity to the reverse-complemented sequence of the other segment. This should not be surprising because heterogeneity in the rate of evolution along the genome leads to non-random clumping of mismatches and, thus, causes overrepresentation of long bubbles^22^. Also, large-scale mutations different from microinversions likely played a significant role in generating bubbles of N > 5. Unfortunately, we do not know a priori how many bubbles are expected to appear in these ways.

While the comparison of the two segments that constitute a bubble can reveal its origin, false-positive microinversions remain a concern. This is because a segment of one genome can be identical or very similar to the reverse-complemented segment of the other genome by sheer accident. If we are dealing with K bubbles, and the probability that a bubble originated by other means but looks like a microinversion is P, we can be confident that every bubble that looks like a microinversion indeed evolved in this way only if P << 1/K, because the expected number of false positives is << 1 in this case.

Under the simplest assumptions, in a bubble of length N, the segment of one genome coincides accidentally with a reverse-complemented segment of the other genome with probability (1/3)^N^, if the bubble contains no matches, or with probability (1/4)^N^, if random matches between the segments are also allowed. Thus, the probability for a bubble of length 10 to look like a product of a microinversion by accident is ~ 10^–5^ for bubbles with no matches or ~ 10^–7^ for bubbles with random matches. Because the total number of bubbles of length 10 and above in the human-chimpanzee genome alignment is only ~ 10^4^, i.e. exceeds those that could have originated by random chance by at least and order of magnitude (Table S2), we assume that practically all bubbles that look like microinversions, are likely definite microinversions. In other words, it seems likely that the minimal length of a bubble in the human-chimpanzee genome alignment that can be confidently interpreted as a microinversion if one of the genome segments (nearly) coincides with the reverse-complemented sequence of the other segment is close to 10.

**Figure 1:**
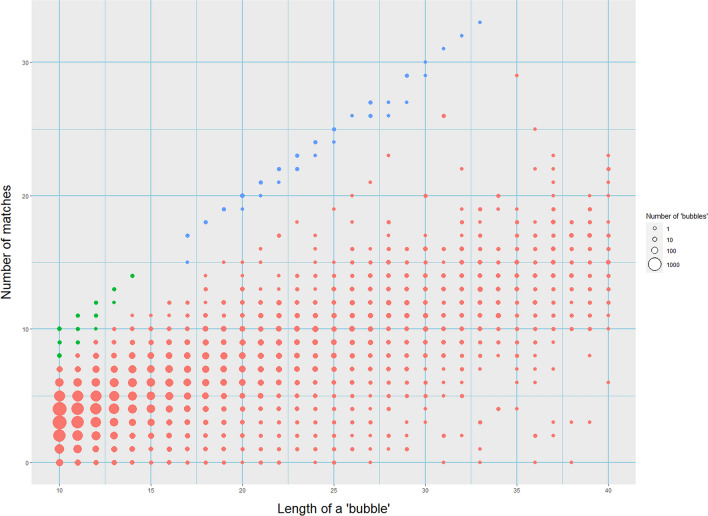
Microinversions in the human-chimpanzee genome alignment. The x-axis corresponds to the length of a “bubble” from 10 to 40 nucleotides; the y-axis corresponds to the number of matches between the reverse complement of chimpanzee DNA sequence and the corresponding human DNA sequence. Blue circles correspond to microinversions that we consider to be definite, green circles—likely microinversions. Sizes of circles corresponds to the number of “bubbles”.

Our data are in agreement with this simple probabilistic reasoning. Table S2 shows bubbles of 5-to 40- nucleotides with different numbers of reverse-complemented matches, while Fig. 1 is a plot representation of the bubbles ranging from 10 to 40 nucleotides. First, vast majority of them (designated by red circles in Fig. 1 or unhighlighted in Table S2) do not possess any excess of such matches and, instead, approximately conform to the binomial distribution with the average number of such matches equal to ~ 1/3 of the length of the bubble. Second, there is a small fraction of bubbles made entirely or almost entirely of reverse-complemented matches (blue and green circles in Fig. 1, and the highlighted diagonal in Table S2). The latter bubbles were likely produced by microinversions while the bubbles of the first kind—by other mechanisms.

As expected, the kind of a bubble becomes clearly recognizable when N is large enough. The two kinds of bubbles form separate modes starting from N ~ 10, and starting from N ~ 15, the chances that a bubble accidentally looks like a product of a microinversion become very low. In contrast, it seems to be impossible to claim with confidence that a bubble with N < 10 was produced by a microinversion, although some of them likely were. To be conservative, we concentrate on microinversions of lengths 15 and above (blue circles in Fig. 1), while the range of N from 10 to 14 (green circles in Fig. 1) is considered by us as a gray area.

We conclude that among a very large number of potential microinversions listed in the Table S2, only 59 events of lengths from 17 to 33 seem to be definite microinversions. In the majority (45) of them, there was a complete match between a human sequence and the corresponding reverse complement chimpanzee’s sequence, while in the remaining 14 cases, there were only one-to-two small differences (mismatches or gaps).

Orangutan genome was used as an outgroup to determine in which lineage, human or chimpanzee, a particular microinversion occurred. Among them, 21 occurred in the human lineages and 28 in the chimpanzee lineage, and 10 could not be assigned due to lack of the outgroup sequences (Table S1).

We did not observe any biases in terms of genome structure or functioning. 31 out of 59 microinversions were located within introns, which is not very different from random expectation because introns occupied ~ 43% of analyzed sequences. Only one gene, *SC5D*, carried a microinversion located in an exon, but it does not overlap with the coding segment and thus does not affect the protein.

The same approach was used to discover microinversions from the chimpanzee—bonobo genome alignment (Table S3). Using the same stringent criteria as for human-chimpanzee analysis, we found 9 microinversions ranging in length from 17 to 25 nucleotides.

### Definite microinversions are flanked by perfect or nearly perfect inverted repeats

**Figure 2:**
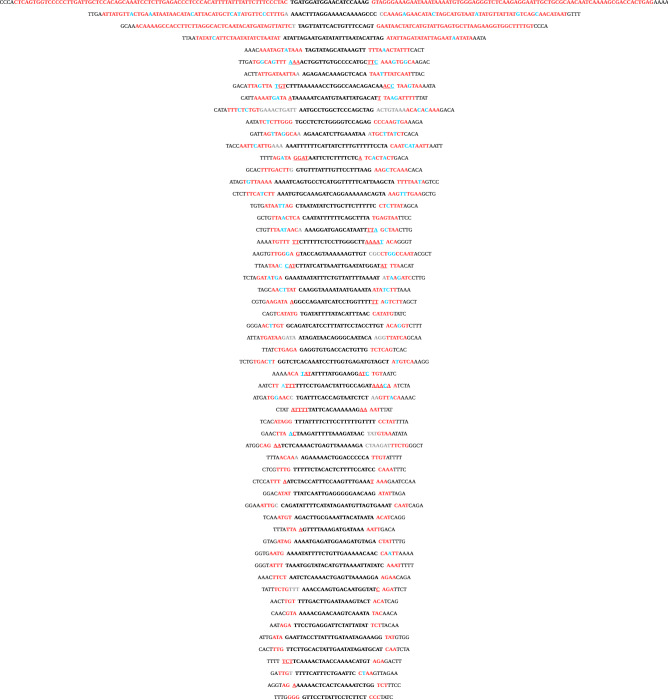
Definite microinversions between human and chimpanzee genomes are always flanked by inverted repeats. Microinversion sequences are shown in bold black font; inverted repeats are shown in red bold font, and their overlaps with microinversions are underlined; mismatches within inverted repeats are shown in blue bold font; sequences separating microinversions from inverted repeats are shown in grey bold font; sequences external to inverted repeats are shown in regular black font.

Notably, each definite microinversion that we found is flanked by inverted repeat sequences (Fig. 2, Table S1). The length of these inverted repeats varies from 3 to 75 nucleotides per flank, 34 of which contain mismatches or small gaps.

**Figure 3:**
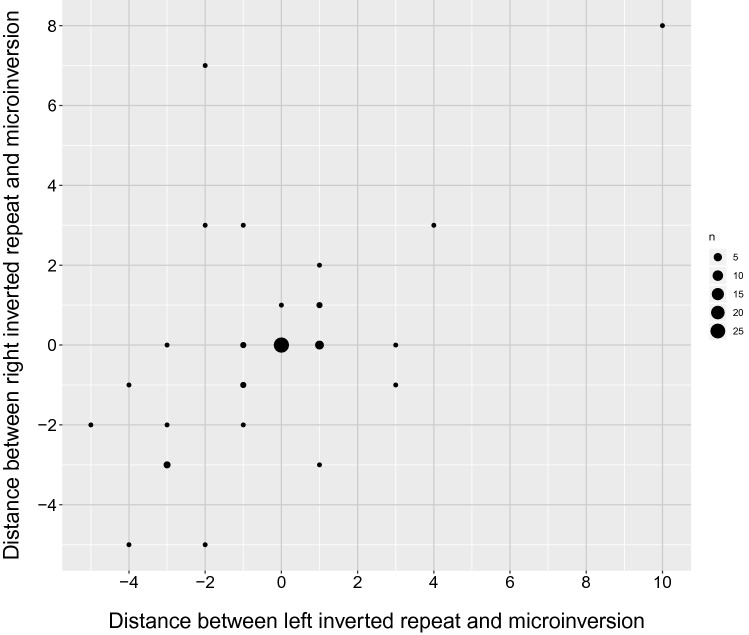
Positions of flanking inverted repeats are correlated with the outer boundaries of microinversions. The x-axis shows the distances in nucleotides between microinversions and inverted repeats on their left; the y-axis shows the distances between microinversions and inverted repeats on their right. Zero value for the left or right repeat means that a repeat is located immediately adjacent to the microinversion; positive value shows that there is a distance between a repeat and the microinversion; negative value shows that a repeat partly overlaps with the microinversion.

With only two exceptions, the inner edge of an inverted repeat is located within − 5 to + 4 nucleotides relative to the outer boundaries of the microinversion. The distances between the outer boundaries of microinversions and the inner edges of inverted repeats are correlated (Pearson’s correlation coefficient, calculated in Fig. 3, is 0.64).

These inverted repeats can be subdivided into three loose classes, based on their length. First, there are relatively long repeats, in which the inverted repeat segment is at least 8 nucleotides or more: 23 of 59 microinversions are surrounded by such repeats. Obviously, these repeats are extremely unlikely to occur by chance, even if we take into account some flexibility in their locations relative to a microinversion.

Secondly, there are 28 repeats of the intermediate length, in which an inverted repeat sequence ranges from 4 to 7 nucleotides. They are also highly unlikely to occur by chance given their proximity to the outer edges of the microinversion.

Finally, there are 8 microinversions that are flanked by only 3 nucleotide-long inverted repeat sequences. Notably, however, 6 of them are directly adjacent to the microinversion edges, the random chance of which is 1/64. In the remaining cases, inverted repeats overlap with the microinversion edges on 2 or 4 nucleotides, respectively. Thus, we conclude that even the shortest inverted repeats flanking microinversions are not spurious as well.

Similarly, all 9 microinversions revealed by comparison between chimpanzee and bonobo genomes are also flanked by inverted repeats ranging from 3 to 10 nucleotides (Table S3).

## Discussion

We found 59 microinversions of lengths from 17 to 33 on the evolutionary path connecting humans and chimpanzees (Fig. 1). These data suggest that during human-chimpanzee divergence definite microinversions appeared at the rate of ~ 2 × 10^–13^ per nucleotide per generation (taking into account the genome size of ~ 3 × 10^9^ nucleotides and assuming that the two lineages together went through ~ 10^6^ generations^23^). Thus, microinversions occur five orders of magnitude less frequently than single-nucleotide substitutions, the rate of which is 10^–8^ per nucleotide per generation rate. We realize that the actual rate of microinversion occurrence must be somewhat higher than we deduced, given that we ignored shorter events and could not analyze hard-to-sequence genomic regions, but it is still orders of magnitude less than the rate of point substitutions, which is not surprising, as a microinversion is obviously a much more complex and improbable event.

Our data are consistent with the constant rate of the origin of microinversions. The human and the chimpanzee lineages accumulated similar numbers of them, which is to be expected because the overall rates of genome evolution of these two lineages are similar^24^. Observing 9/59 times less microinversions on the path connecting chimpanzees and bonobos agrees well with this path being ~ 4 times shorter than that between humans and chimpanzees^25^.

In 45 of the 59 microinversion, the human genome segment precisely coincided with the reverse-complemented chimpanzee genome segment, while in the remaining 14 there is one or two differences, either a mismatch or a gap. At the moment of its occurrence, a microinversion can be pure, if the replacement of the genome segment with the reverse-complemented sequence is not accompanied by any other mutations. Alternatively, a microinversion can be impure to start with, if its occurrence was accompanied by other, small-scale mutations, as it is usually the case for long inversions^8,15,26^. Our data indicate that, in contrast to long inversions, definite microinversions are usually born pure.

Indeed, the divergence between human and chimpanzee genomes is ~ 1%. Because the characteristic length of our microinversions is ~ 20 nucleotides, we expect, if small-scale differences within microinversions emerge independently, that 1/5 of microinversions carry 1 mismatch, 1/25 carry 2 mismatches, etc. Our data conform to these expectations. Moreover, among the 3 small-scale differences within the segments of the human-chimpanzee alignment that correspond to a microinversion that can be attributed to a particular lineage, 6 occurred not in the lineage where the microinversion happened, but in the other one (Table S1).

Note that the number of microinversions that we found is much smaller than that in a recent study of Walker et al.^27^. This is because our approaches are radically different. Walker et al. strived to find all plausible microinversions by employing hidden Markov models for this purpose. Their set contains over 4000 microinversions, some of which are as short as 6 nucleotides. Obviously, even if such a short segment of the human-chimpanzee alignment contains sequences that are exactly reverse-complementary to each other, one cannot be certain that it, indeed, is the result of a microinversion, although it is the most likely explanation. In contrast, we tried to avoid any possibility of false positive events and identified only definite microinversions relying on simple probabilistic arguments described in “Results” section. Therefore, we only analyzed microinversion longer than 15 nucleotides, for which the probability of false discovery is vanishingly small (Fig. 1).

All definite microinversions that we detected appeared to be flanked by perfect or nearly perfect inverted repeats. Roughly 39% of these inverted repeats are relatively long (> 8 nucleotides per flank), 50% are of an intermediate length (4 to 8 nucleotides per flank) and the remaining 11% are short (3 nucleotides per flank). Most importantly, the inner edges of these inverted repeats juxtapose with the boundaries of the microinversions in practically every case (Figs. 2, 3). We conclude that the combination of the inverted repeat lengths and precise location makes their chance occurrence at microinversions highly unlikely.

Thus, the following two observations are principal when considering a mechanism(s) responsible for the microinversion formation. First, clean origin of most our microinversions implies that whatever mechanism caused this rearrangement, the inverted segment was copied precisely. Second, since an inverted segment is surrounded by inverted repeats in all the cases, they are most likely involved in the process.

The conventional wisdom about the inversion formation is that a DNA segment flanked by two homologous DNA sequences in an inverted orientation would flip upon recombination between these flanking sequences^28^. Two branches of recombination were shown to be involved. Homologous recombination (HR) between long inverted DNA elements, for example two non-LTR retrotransposons, are known to cause inversions^29^. Note, however, that the minimal homology length for HR in eukaryotes is somewhere between 50 and 250 base pairs^30,31^. Since only two of our 59 microinversions are flanked by the repeats exceeding this 50 bp, we can effectively rule out this mechanism. On the opposite end of the spectrum is site-specific recombination, where specialized DNA recombinases carry out DNA strand exchange between short inverted repeat (IR) sequences resulting in inversions^32^. While this mechanism is mostly used by prokaryotes, it is not without precedence in humans. For example, Rag1/Rag2 recombinase makes inversions during V(D)J recombination of immunoglobulin light chain genes^33^. We don’t think, however, that site-specific recombination is the cause of microinversions. One counterargument is that given the lack of sequence similarity between IRs flanking different microinversions, one would have to assume the involvement of multiple site-specific recombinases. Even more importantly, two IRs need to be aligned in a parallel way for the recombination to proceed, thus, the central DNA segment must form a loop^34^. This is physically impossible for the microinversions of our size (< 33 bp), given that the persistence length of DNA corresponds to ~ 145 base pairs^35^.

Perfect or imperfect inverted repeats were shown to induce template switching events during DNA replication. In a nutshell, the unique sequence composition of the inverted repeat allows DNA polymerase to jump between its halves situated on either template or nascent DNA strands leading to mutations and genome rearrangements^18^. It was suggested that template switching may account for the inversions of DNA segments flanked by inverted repeats^7,19,20,36^. Most recently, Walker et al.^27^ described a method for modelling and detecting mutations that appeared upon template switching events, which identified thousands of such events (4017 unique cases) in the great apes genomes. Various genome rearrangements of different lengths, including thousands of potential microinversions, were detected among those template-switching events. Note, however, that the majority of these microinversions were shorter than 15 nucleotides, many as short as 1 nt-long, which, as discussed above, are hard to ascertain as definite inversions.

**Figure 4:**
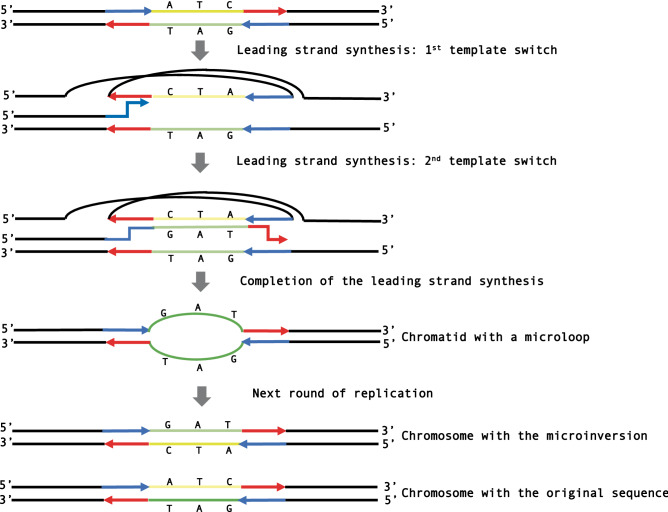
Template-switching model for the formation of microinversions. Complementary strands of a DNA segment to undergo inversion are shown as yellow and green lines. Complementary strands of flanking inverted repeats are shown as red and blue arrows; the tips of these arrows designate 3′ ends of inverted repeats (see text for details).

For the microinversion to occur via template switching, two template switches at inverted repeats during DNA replication must occur as discussed in Refs.^7,27,36^. Figure 4 shows how two template-switching events during DNA replication can create a microinversion. First, the leading DNA polymerase jumps from the first half of the flanking IR in the leading strand template to the identical sequence in the fold-back lagging strand template. Note that this jump is facilitated by the trombone-like configuration of the DNA strands at the replication fork^37^. Second, the leading DNA polymerase synthesizes the reverse complement of its anticipated template until it reaches the second half of the IR. Third, the DNA polymerase jumps to the identical sequence in the leading strand template resuming normal replication. This sequence of events gives rise to a newly synthesized DNA molecule with a microloop consisting of two inverted strands at the site of future microinversion. After the second round of replication, two DNA molecules arise, one of which contains the microinversion.

While similar models were discussed in earlier studies^7,19,27,36^, they have two potential caveats. First, since two jumps of DNA polymerase must occur within a half of an inverted repeat, one would expect it to be sufficiently long. It is hard to imagine how the two jumps could realistically happen within a sequence that is only 3 or 4 nt-long. The second problem is that template switching of imperfect inverted repeats is known to convert them into perfect ones^38^. We believe, therefore, that the template-switch model could explain the formation of those definite microinversions that are flanked by longer, perfect inverted repeats (such as microinversions in the upper part of Fig. 2), while it is hard to apply to explain the formation of microinversions that are flanked by very short IRs, or longer, but imperfect IRs.

As an alternative, it was previously discussed that inversions can result from an incorrect repair of two double-strand breaks (DSBs) formed within flanking inverted repeats via non-homologous end-joining (NHEJ)^9^. We don’t think, however, that this scenario applies to our microinversions, since formation of two DSBs separated by ~ 20 nucleotides is highly unlikely even in a strong DNA damaging environment such as ionizing radiation. A more likely scenario—repair of a single DSB positioned proximal or distal to a hairpin-forming sequence—was shown to form foldback inversions (a.k.a. inverted duplications)^39^, that are principally different from the microinversions described here.

**Figure 5:**
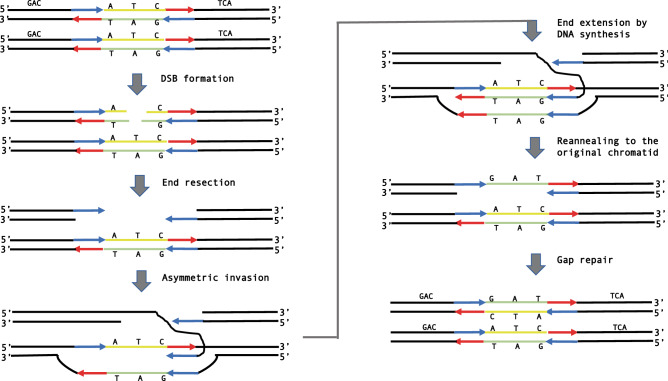
Microhomology-mediated BIR model for the microinversion formation. Complementary strands of a DNA segment to undergo inversion are shown as yellow and green lines. Complementary strands of flanking inverted repeats are shown as red and blue arrows; the tips of these arrows designate 3′ ends of inverted repeats. See text for details.

We propose a new mechanism for the microinversion formation during DSB repair that is applicable to all definite microinversions found by us (Fig. 5). The trigger is a DSB in the region to undergo inversion, which is present in one sister chromatid in the G2 phase of the cell cycle. Subsequent end-resection would expose the flanking inverted repeat in a single-stranded state. This is followed by an asymmetric invasion of the IR from the left flank of the inverting segment into the sister chromatid, such that it pairs with the right flank IR. The invaded DNA strand is then extended to the left flank IR followed by its reannealing with the original chromatid. The following gap repair synthesis completes the inversion process. Note that in contrast to the template-switching model, our DSB repair model does not involve the presence of single-stranded loop-outs, that are known to be hypermutagenic^40^. Consequently, the DSB repair model is consistent with the lack of mutations in the inverting segments characterized in this study.

At a first glance, this model is reminiscent of the synthesis-dependent strand annealing (SDSA), which leads to the formation of non-crossover products during homologous recombination^41^. There are two significant differences, however. First, the invasion and pairing occur between IRs on the opposite sides of the break, in contrast with regular SDSA, where DNA segments on the same side of the break are being paired. We believe that this asymmetric invasion should not be a problem for microinversions, the length of which is well within the limits of single-stranded DNA flexibility^42^. Second, in canonical SDSA the region of homology at the invading end should be significantly longer (> 50 nucleotides)^41^ than IRs flanking our microinversions. Altogether, our model is much more like an alternative pathway of DSB repair called break-induced replication (BIR)^43^, more specifically microhomology-mediated BIR^44,45^. While initially proposed to explain genome rearrangements observed in patients with hereditary diseases^44^, microhomology-mediated BIR was recently characterized experimentally, and the microhomologies required for invasion and strand extension were comparable with our flanking IRs^46,47^. We believe therefore, the model presented in Fig. 5 could satisfactorily explain the majority of the definite microinversion events observed in this study.

Of note, while DNA repair events leading to the microinverstion formation shown in Fig. 5 take place between sister chromatids in the G2 phase prior to mitosis, we speculate that similar microhomology-mediated BIR events could also happen between homologous chromosomes in meiosis, resulting in non-crossover products with microinversions. Finally, this model can be particularly applicable to multiple microinversions observed in cancer genomes^16^, given that DSBs are among the main drivers of the genome instability causing cancer development^48^.

## Methods

Human (hg38) and common chimpanzee (panTro5) genomes (http://hgdownload.soe.ucsc.edu/goldenPath/hg38/bigZips/hg38.fa.gz; http://hgdownload.soe.ucsc.edu/goldenPath/panTro5/bigZips/panTro5.fa.gz) and their pairwise alignment (http://hgdownload.cse.ucsc.edu/goldenPath/hg38/vsPanTro5/), as well as data for chimpanzee (panTro4) and bonobo (panPan2) (genomes https://hgdownload.soe.ucsc.edu/goldenPath/panTro4/bigZips/panTro4.fa.gz; https://hgdownload.soe.ucsc.edu/goldenPath/panPan2/bigZips/panPan2.fa.gz and alignment; http://hgdownload.soe.ucsc.edu/goldenPath/panPan2/vsPanTro4/), were obtained from the UCSC Genome Browser. Annotation for human genome (http://hgdownload.soe.ucsc.edu/goldenPath/hg38/database/knownCanonical.txt.gz) and data on simple repeats (http://hgdownload.soe.ucsc.edu/goldenPath/hg38/database/simpleRepeat.txt.gz) were also obtained from the same source.

The alignment of human and chimpanzee genomes available from the UCSC Genome Browser consists of alternating regions aligned by LASTZ^49^ and regions that were left unaligned. This alignment, which represented the point of departure for our analysis, did not attempt to discover microinversions, because reverse-complemented sequence segments were not considered. Thus, a microinversion usually was represented by a “bubble”, a pair of unaligned genome segments.

However, in some cases the quality of the alignment of a genome segment with the corresponding reverse-complemented sequence was high enough to include these “false” alignments into the UCSC Genome Browser, as exemplified by this case:

----TGTCTTTAAAAAACCTGGCCA--------ACAGACAAACC
- GGTTTGTCTGT--------TGGCCAGGTTTTTTAAAGACA----

Here, the reverse-complemented chimpanzee genome segment matches the human genome segment precisely, suggesting that a microinversion took place. However, the two genome segments can also be aligned, and this alignment was presented by the UCSC Genome Browser. That is why it is necessary to treat not only genome segments that were left unaligned but also loose segments of the alignment as possible microinversions.

In the human-chimpanzee genome alignment available from the UCSC Genome Browser, only ~ 80% of the genomes are aligned to each other. We first refined this alignment by aligning the corresponding human and chimpanzee genome segments of lengths above 15 nucleotides that were left unaligned, as long as the difference between their lengths did not exceed 2%. Biopython package pairwise2 (with a matrix BLOSUM62 and penalties for gap opening and extending − 10 and − 0.5, respectively) was used for this purpose.

After this, we ascertained microinversions from the refined alignment as follows. We first searched for suspicious “bubbles” in it, flanked by segments where the alignment is reliable and unambiguous. Each mismatch within an alignment was treated as the possible beginning of a bubble. We compared regions of alignments that started from a mismatch M and assigned a penalty of − 0.5 for a match and + 1 for a mismatch or a gap (these parameters resulted in the maximal number of detected microinversions), until the total score became negative. After this, we defined a potential bubble as the segment of the alignment from the initial mismatch M to the position where the total score was maximal. A potential bubble was considered to be real if the following conditions were met: its length was at least 5 positions on the alignment, and it was flanked by alignment segments of lengths above 100 nucleotides that were of a good quality (without tracks of gaps or simple repeats). Search for the next bubble commenced from the next mismatch after the end of the previous bubble, if one was detected, or, otherwise, after the mismatch M.

We ignored bubbles in which the genome segment of at least one of the two species was at least partially masked by the RepeatMasker, as well as those located in poorly assembled regions of genomes (“Unknown” chromosomal regions in at least one species). Every remaining bubble was investigated for the possible presence of a microinversion. We made the reverse complement of the chimpanzee genome segment which resides within a bubble and aligned it to the corresponding human genome segment by the Smith-Waterman algorithm^50^ using Biopython package pairwise2 as described above. Those bubbles for which the resulting alignment contained less than 50% of gaps were treated as potential microinversion, although only a small proportion of them are likely to be real microinversions.

## Supplementary Information

Supplementary Tables.

## Data Availability

Data supporting the findings of this work are available within the paper and its Supplementary Information files.
