# Supplementary material for: Characteristics and possible mechanisms of formation of microinversions distinguishing human and chimpanzee genomes
Source: Sci Rep. 2022 Jan 12;12:591. doi: 10.1038/s41598-021-04621-w (PMC8755829; doi:10.1038/s41598-021-04621-w)
Supplement: Supplementary file 1 — Supplementary Tables. [file 41598_2021_4621_MOESM1_ESM.pdf]

Table S1. Detailed description of true microinversions between human and chimpanzee segments.

chr hg38	Start coordinate microinversion hg38	End coordinate microinversion hg38	Microinversion sequence hg38	Reverse complement microinversion sequence panTro5	Length of realigned fragment	Number of differences with reverse complement seq	Sequence similarity with orangutan	Location in hg38	Length left repeat	Length right repeat	Mismatches number	Gaps number	Distance to microinv for left repeat	Distance to microinv for right repeat
chr1	177645258	177645278	GTGTTTATTTGTTCCTTTAAG	GTGTTTATTTGTTCCTTTAAG	21	0	NA	NA	8	9	0	1	1	0
chr10	117511034	117511054	TAGTTATTCACTGTTTCCAGT	TAGTTATTCACTGTTTCCAGT	21	0	NA	Intron EMX2OS	43	43	0	0	1	0
chr10	25414785	25414801	AGAGAACAAAGCTCACA	AGAGAACAAAGCTCACA	17	0	Pan troglodytes	Intron LINC02624	11	12	0	1	1	0
chr10	15787022	15787047	TTCTTTTTCTCCTTGGGCTTAAAAT	TTCTTTTTCTCCTTGGGCTTAAAAT	25	0	Pan troglodytes	Intron MINDY3	7	8	0	1	-2	-5
chr10	20600922	20600943	ATTTTTATTCACAAAAAAGAA	ATTTTTATTCACAAAAAAGAA	21	0	Pan troglodytes	NA	5	5	0	0	-5	-2
chr10	106158291	106158308	AGAAAAACTGGACCCCCA	AGAAAAACTGGACCCCCA	18	0	Homo sapiens	Intron GPR158	4	4	0	0	1	0
chr11	20511879	20511896	TAGTATAGCATAAAGTT	TAGTATAGCATAAAGTT	17	0	Pan troglodytes	Exon SC5D	12	12	1	0	0	0
chr11	121307997	121308017	TTTGACTTGAATAAAGTACT	TTTGACTTGAATAAAGTACT	20	0	Pan troglodytes	NA	3	3	0	0	0	0
chr12	78474059	78474082	TTTATTTTCTTCCTTTTTGTTTT	TTTATTTTCTTCCTTTTTGTTTT	23	0	Homo sapiens	NA	5	5	0	0	0	0
chr14	25872261	25872281	TGATATTTTATACATTTAAC	TGATATTTTATACATTTAAC	20	0	Homo sapiens	NA	6	6	0	0	0	0
chr14	54355965	54355986	ACTAAGATTTTTAAAGATAAC	ACTAAGATTTTTAAAGATAAC	21	0	NA	NA	5	4	0	1	-2	3
chr15	32997355	32997382	GCAGATCATCCTTTATTCCTACCTTGT	GCAGATCATCCTTTATTCCTACCTTGT	27	0	Pan troglodytes	Intron FMN1	6	6	1	0	0	0
chr16	12616697	12616716	AAAACGAACAAGTCAAATA	AAAACGAACAAGTCAAATA	19	0	Homo sapiens	NA	3	3	0	0	0	0
chr17	3155078	3155101	TTTTTCTACACTCTTTTCCATCC	TTTTTCTACACTCTTTTCCATCC	23	0	Homo sapiens	Intron LOC100288728	4	4	0	0	0	0
chr19	56780515	56780534	GTACCAGTAAAAAAGTTGT	GTACCAGTAAAAAAGTTGT	19	0	Homo sapiens	Intron ZIM2	7	8	1	1	-1	3
chr2	67645552	67645571	ATAGATAACAGGGCAATACA	ATAGATAACAGGGCAATACA	20	0	NA	NA	6	6	0	0	4	3
chr2	36568454	36568479	AATCTACCATTTCCAAGTTTGAAAT	-ATCTACCATTTCCAAGTTTGAAAT	25	1	Homo sapiens	Intron FEZ2	4	4	0	0	-1	-1
chr2	147936480	147936501	TTCCTGAGGATTCTATTATAT	TTCCTGAGGATTCTATTATAT	21	0	Homo sapiens	Intron ORC4	3	3	0	0	0	0
chr20	8732731	8732760	ATATTAGAATGATATATTTAATACATTAG	ATATTAGAATAATATATTTAATATATTAG	29	2	Homo sapiens	Intron PLCB1	25	25	1	0	0	0
chr3	7808863	7808884	AAACTTTAGGAAAACAAAAGCCC	GAACTTTAGGAAAACAAAAGCCC	23	1	Homo sapiens	NA	51	51	6	0	1	1
chr3	70947508	70947528	AATGCCTGGCTCCCAGCTAG	AATGCCTGGCTCCCAGCTAG	20	0	Pan troglodytes	Intron FOXP1	9	9	2	0	10	8
chr3	25866529	25866562	AAAATCAGTGCCTCATGGTTTTTCATTAAGCTA	AAAATCAGTGCCTCATGGTTTTTCATTAAGCTA	33	0	Homo sapiens	Intron NAALADL2	8	8	1	0	0	0
chr3	30778489	30778518	AAATGTGCAAAGATCAGGAAAAAACAGTA	AAATGTGCAAAGATCAGGAAAAAACAGTA	29	0	Homo sapiens	NA	8	9	1	1	0	0
chr3	175798858	175798886	CATCTTATCATTAAATTGAATATGGATAT	CATCTTATCATTAAATTGAATATGGATAT	29	0	Homo sapiens	Intron LINC00692	7	5	0	2	-3	-2
chr3	70967772	70967790	GAGGTGTGACCACTGTTG	GAGGTGTGACCACTGTTG	18	0	Pan troglodytes	Intron GADL1	6	6	0	0	0	0
chr3	44156109	44156139	GGTCTCACAAATCCTTGGTGAGATGTAGCT	GGTCTCACAAATCCTTGGTGAGATGTAGCT	30	0	Homo sapiens	Intron GADL1	6	6	1	0	0	0
chr3	30768485	30768507	TTATCAATTGAGGGGGAACAAG	TTATCAATTGAGGGGGAACAAG	22	0	Homo sapiens	NA	4	4	0	0	0	0
chr3	25097445	25097475	CAGATATTTTCATATAGAATGTTAGTGAAAT	CAGATATTTTCATATAGAATGTTAGTGAAAT	31	0	NA	Intron RARB	4	4	0	0	1	0
chr3	30820209	30820236	GAATTACCTTATTTGATAATAGAAAGG	GAATTACCTTATTTGATAATAGAAAGA	27	1	Homo sapiens	Intron GADL1	3	3	0	0	0	0
chr4	77203811	77203835	AAAACTGGTTGTGCCCCATGCTTC	AAAACTGGTTGTGCCCCATGCTTC	24	0	Pan troglodytes	NA	12	12	3	0	-3	-3
chr4	181206447	181206466	TATATTTTATGGAAGGATC	TATATTTTATGGAAGGATC	19	0	Homo sapiens	NA	6	6	1	0	-3	-3
chr4	180829078	180829100	AGACTTGCGAAATTACATAATA	AGACTTGCAAAATTACATAATA	22	1	Homo sapiens	NA	4	4	0	0	0	0
chr4	127543746	127543773	TTCTTGCACTATTGAATATAGATGCAT	TTCTTGCACTATTGAATATAGATGCAT	27	0	Pan troglodytes	NA	3	3	0	0	0	0
chr5	128172010	128172042	TGTCTTTAAAAAACCTGGCCAACAGACAAACC	TGTCTTTAAAAAACCTGGCCAACAGACAAACC	32	0	Pan troglodytes	Intron SLC12A2	10	10	2	0	-3	-3
chr5	133904341	133904361	TGCCTCTCTGGGGTCCAGAG	TGCCTCCCTGGGGTCCAGAG	20	1	Pan troglodytes	NA	9	9	1	0	0	0
chr6	44468404	44468425	TGATGGATGGAACATCCAAAG	TGATGGATGGAACGTCCAAAG	21	1	Homo sapiens	NA	75	75	0	0	0	0
chr6	130415991	130416017	ATAAAAATCAATGTAATTATGACATT	ATAAAAATCAATGTAATTATGACATT	26	0	NA	Intron TMEM200A	10	10	2	0	-1	-1
chr6	20824933	20824956	CTAATATATCTTGCTTCTTTTTC	CTAATATATCTTGCTTCTTTTTC	23	0	NA	NA	8	7	1	1	0	0
chr6	9050537	9050562	GAAATAATATTTCTGTTATTTTAAAAT	GAAATAATATTTCTGTTATTTTAAATT	27	1	NA	NA	7	7	2	0	0	1
chr6	23418408	23418428	CAAGGTAAAATAATGAAATA	CAAGGTAAAATAATGAAATA	20	0	Pan troglodytes	Intron CDKAL1	7	7	2	0	0	0
chr6	40158125	40158149	AGGCCAGAATCATCCTGGTTTTTT	AGGCCAGAATCATCCTGGTTTTT-	24	1	Homo sapiens	NA	7	8	0	1	-1	-2
chr6	153859867	153859897	ATTTTTTCCTGAACTATTGCCAGATAAACA	ATTTTTTCCTGAACTATTGTCAGATAAACA	30	1	Homo sapiens	NA	6	6	1	0	-4	-5
chr6	70841027	70841044	AGTTTTAAAGATGATAAA	AGTTTTAAAGATGATAAA	18	0	Pan troglodytes	NA	4	4	0	0	-1	0
chr6	40108267	40108289	AAAATGAGATGGAAGATGTAGA	AAAATGAGATGGAAGATGTAGA	22	0	Pan troglodytes	NA	4	4	0	0	0	0
chr6	146801071	146801096	AAAATATTTTCTGTTGAAAAACAAC	AAAATATTTTCTGTTGAAAAACAAC	25	0	Homo sapiens	NA	4	5	0	1	0	0
chr6	131149179	131149202	TCTTCAAAACTAACCAAAACATGT	TCTTCAAAACTAACCAAAACATGT	24	0	Homo sapiens	Intron SMAP1	3	3	0	0	-3	0
chr6	40080444	40080460	TTTTCATTTCTGAATTC	TTTTCATTTCTGAATTC	17	0	Homo sapiens	Intron ADGB	3	4	0	1	1	0
chr7	3642662	3642677	AGAACATCTTGAAATAA	-GAACACCTTGAAATAA	17	2	NA	Intron SDK1	9	9	2	0	1	1
chr7	107939937	107939956	CAATATTTTTTCAGCTTTA	CAATATTTTTTCAGCTTTA	19	0	Pan troglodytes	Intron NXPH1	8	7	0	1	0	0
chr7	8746484	8746503	AAAGGATGAGCATAATTTTA	AAAGGATGAGCATAATTTTA	20	0	Pan troglodytes	Intron COL26A1	8	8	1	2	1	-3
chr7	101368887	101368910	-AAAAAACTCACTCAAAATCTGG	AAAAAAACTCACTCAAAATCTGG	23	1	Pan troglodytes	intron LAMB1	3	3	0	0	-1	0
chr9	92252310	92252336	AAATTTTTTCATTATCTTTGTTTTTCCTA	AAATTTTTTCATTATCTTTGTTTTTCCTA	29	0	Pan troglodytes	Intron LINGO2	9	11	1	2	3	0
chr9	79214316	79214335	GGATAATTCTCTTTTCTCA	GGATAATTCTCTTTTCTCA	19	0	NA	Intron IARS1	9	9	0	2	-4	-1
chr9	3878519	3878539	TGATTTCACCAGTAATCTCT	TGATTTCACCAGTAATCTCT	20	0	Homo sapiens	NA	6	6	1	0	1	2
chr9	28044305	28044333	TAAATGGTATACATGTTAAAATTATATC	TAAATGGCATACATGTTAAAATTATATG	28	2	Pan troglodytes	Intron GLIS3	4	4	0	0	0	0
chr9	78721530	78721547	GTTCCTTATTCCTCTTCT	GTTCCTTATTCCTCTTCT	18	0	Homo sapiens	NA	3	3	0	0	0	0
chrX	32880626	32880650	AATCTCAAAACTGAGTTAAAAAGA	AATCTCAAAACTGAGTTAAAAAGA	24	0	Homo sapiens	NA	5	5	0	0	-2	7
chrX	32738672	32738700	AATCTCAAAACTGAGTTAAAAGGA	AATCTCAAAACTGAGTTAAAAAGA	28	1	Homo sapiens	Intron DMD	4	4	0	0	0	0
chrX	25873876	25873897	AAACCAAGTGACAATGGTATC	AAACCAAGTGACAATGGTATC	21	0	Pan troglodytes	Intron DMD	4	4	0	0	3	-1

Number of matches between the reverse complement of chimpanzee DNA sequence and the corresponding human DNA sequence

Length of a "bubble" (rows)

	0	1	2	3	4	5	6	7	8	9	10	11	12	13	14	15	16	17	18	19	20
5	12029 / 13	16205 / 40	8627 / 16	2898 / 1	586 / 0	194 / 0															
6	3694 / 16	6281 / 13	6129 / 22	3785 / 7	1520 / 3	263 / 1	80 / 1														
7	2171 / 5	3229 / 12	4526 / 10	3038 / 6	1620 / 3	421 / 1	116 / 0	34 / 0													
8	647 / 0	1611 / 2	2707 / 10	2355 / 11	1700 / 6	547 / 2	141 / 0	13 / 0	18 / 1												
9	275 / 1	813 / 4	1493 / 9	1534 / 8	1068 / 10	455 / 2	131 / 1	26 / 0	4 / 0	3 / 1											
10	114 / 1	378 / 1	822 / 6	1200 / 2	1161 / 12	567 / 4	253 / 1	57 / 0	12 / 0	3 / 0	7 / 0										
11	61 / 0	239 / 4	545 / 4	830 / 5	815 / 5	559 / 3	260 / 3	71 / 1	10 / 1	2 / 1	2 / 0	4 / 2									
12	26 / 2	102 / 1	303 / 1	501 / 2	661 / 4	565 / 6	283 / 2	118 / 2	53 / 1	9 / 0	1 / 0	3 / 1	2 / 1								
13	14 / 10	55 / 2	181 / 1	277 / 2	495 / 1	417 / 5	331 / 11	129 / 3	42 / 0	8 / 1	6 / 0	0 / 0	1 / 0	1 / 1							
14	10 / 0	34 / 0	76 / 1	177 / 0	339 / 1	326 / 2	311 / 0	193 / 5	89 / 3	20 / 0	7 / 0	1 / 0	0 / 0	0 / 0	1 / 1						
15	5 / 0	14 / 0	54 / 0	128 / 1	238 / 1	278 / 4	245 / 3	162 / 1	95 / 1	26 / 0	8 / 0	1 / 0	0 / 0	0 / 0	0 / 0	0 / 0					
16	9 / 0	19 / 0	37 / 0	77 / 0	127 / 0	201 / 6	216 / 2	166 / 2	105 / 1	45 / 0	12 / 0	1 / 0	5 / 0	0 / 0	0 / 0	0 / 0	0 / 0				
17	2 / 0	13 / 0	17 / 0	37 / 0	107 / 1	147 / 0	175 / 1	137 / 3	100 / 1	33 / 2	9 / 1	3 / 1	2 / 0	0 / 0	0 / 0	0 / 1	0 / 0	1 / 2			
18	2 / 0	9 / 0	14 / 0	32 / 0	74 / 0	103 / 0	143 / 3	141 / 1	115 / 4	57 / 0	35 / 0	13 / 0	1 / 0	2 / 1	1 / 0	0 / 0	0 / 0	0 / 0	1 / 3		
19	6 / 0	7 / 0	16 / 0	18 / 0	29 / 0	66 / 0	105 / 3	99 / 1	121 / 1	43 / 1	36 / 1	5 / 0	10 / 0	0 / 0	0 / 0	0 / 1	0 / 0	0 / 0	0 / 0	5 / 0	
20	1 / 0	2 / 0	8 / 0	8 / 0	30 / 1	44 / 8	81 / 0	108 / 1	99 / 4	64 / 4	44 / 1	11 / 0	9 / 1	6 / 1	1 / 0	1 / 0	0 / 0	0 / 0	0 / 0	1 / 0	5 / 2
21	2 / 1	4 / 1	3 / 0	6 / 0	16 / 0	43 / 0	55 / 0	63 / 0	71 / 2	63 / 1	29 / 0	23 / 0	7 / 0	1 / 0	0 / 0	0 / 1	1 / 0	0 / 0	0 / 0	0 / 0	1 / 0
22	2 / 1	4 / 0	6 / 0	4 / 0	15 / 0	12 / 0	42 / 0	61 / 3	68 / 9	70 / 3	41 / 0	23 / 0	15 / 2	1 / 0	1 / 0	0 / 0	0 / 0	2 / 0	0 / 0	0 / 0	0 / 0
23	0 / 0	2 / 0	5 / 0	4 / 0	11 / 0	12 / 0	18 / 1	34 / 1	53 / 0	37 / 0	43 / 0	28 / 0	12 / 1	5 / 0	3 / 0	0 / 0	0 / 0	0 / 0	0 / 1	0 / 0	0 / 0
24	1 / 0	2 / 0	2 / 0	2 / 0	6 / 0	7 / 1	11 / 1	34 / 1	28 / 1	43 / 0	63 / 1	25 / 0	11 / 2	1 / 0	2 / 0	1 / 0	1 / 0	1 / 0	0 / 0	0 / 0	0 / 0
25	4 / 0	2 / 0	2 / 0	4 / 0	4 / 0	5 / 0	15 / 1	25 / 2	34 / 1	50 / 1	22 / 1	21 / 0	13 / 0	8 / 1	4 / 0	1 / 0	0 / 0	0 / 0	0 / 0	1 / 0	0 / 0
26	0 / 0	2 / 0	1 / 0	3 / 0	2 / 0	2 / 0	11 / 0	14 / 0	22 / 1	28 / 0	37 / 1	25 / 2	20 / 0	9 / 0	4 / 0	4 / 0	2 / 0	0 / 0	1 / 0	0 / 0	0 / 1
27	1 / 0	1 / 0	1 / 0	2 / 0	1 / 0	1 / 0	4 / 0	12 / 0	14 / 2	20 / 0	21 / 0	19 / 1	16 / 0	12 / 1	10 / 0	5 / 0	1 / 0	0 / 0	0 / 0	0 / 0	0 / 0
28	0 / 0	1 / 0	4 / 0	0 / 0	2 / 0	3 / 0	4 / 0	12 / 0	7 / 1	12 / 1	14 / 2	25 / 0	17 / 1	13 / 1	9 / 0	10 / 0	4 / 0	1 / 0	2 / 0	0 / 0	0 / 0
29	0 / 0	2 / 0	1 / 0	1 / 0	0 / 0	2 / 0	4 / 0	12 / 0	10 / 1	7 / 1	15 / 1	19 / 0	16 / 5	8 / 0	7 / 0	3 / 0	5 / 0	0 / 0	0 / 0	0 / 0	0 / 0
30	0 / 0	0 / 0	0 / 0	1 / 0	1 / 0	3 / 0	2 / 0	1 / 0	14 / 0	11 / 0	22 / 2	24 / 0	13 / 0	16 / 0	11 / 0	4 / 0	9 / 0	1 / 0	0 / 0	0 / 0	2 / 0
31	1 / 0	0 / 0	4 / 0	0 / 0	0 / 0	1 / 0	1 / 0	1 / 0	7 / 0	7 / 1	7 / 0	14 / 0	4 / 0	11 / 0	12 / 0	1 / 0	5 / 0	0 / 0	0 / 0	0 / 0	0 / 0
32	0 / 0	0 / 0	1 / 0	0 / 0	0 / 0	2 / 0	1 / 0	2 / 0	3 / 2	15 / 0	8 / 0	11 / 0	10 / 0	9 / 0	10 / 1	9 / 0	8 / 0	1 / 0	6 / 0	1 / 0	0 / 0
33	1 / 0	1 / 0	0 / 0	2 / 0	0 / 0	0 / 0	0 / 0	1 / 0	6 / 0	5 / 0	5 / 8	9 / 0	16 / 0	18 / 0	10 / 0	6 / 1	3 / 1	3 / 0	1 / 0	1 / 1	0 / 0
34	0 / 0	0 / 0	0 / 0	0 / 0	2 / 0	0 / 0	0 / 0	0 / 0	1 / 0	2 / 0	2 / 0	15 / 2	14 / 0	10 / 0	9 / 0	3 / 0	3 / 0	0 / 0	0 / 0	1 / 0	3 / 0
35	0 / 0	0 / 0	0 / 0	0 / 0	1 / 0	0 / 0	1 / 0	1 / 0	2 / 0	2 / 0	2 / 0	3 / 0	4 / 0	6 / 1	8 / 0	7 / 1	4 / 0	3 / 2	2 / 0	1 / 0	0 / 0
36	1 / 0	0 / 0	2 / 0	0 / 0	0 / 0	0 / 0	1 / 0	1 / 0	1 / 0	2 / 1	4 / 0	4 / 0	7 / 1	4 / 1	7 / 0	2 / 0	5 / 0	4 / 1	1 / 0	0 / 0	0 / 0
37	0 / 0	0 / 0	1 / 0	1 / 0	0 / 0	0 / 0	0 / 0	1 / 0	0 / 0	1 / 0	3 / 0	3 / 0	2 / 1	6 / 0	7 / 0	7 / 0	9 / 0	4 / 0	7 / 1	2 / 0	1 / 0
38	1 / 0	0 / 0	0 / 0	1 / 0	0 / 0	0 / 0	0 / 0	0 / 0	0 / 0	0 / 0	2 / 0	2 / 0	1 / 0	5 / 0	6 / 1	6 / 0	6 / 0	8 / 0	2 / 0	0 / 0	0 / 0
39	0 / 0	0 / 0	0 / 0	1 / 0	0 / 0	0 / 0	0 / 0	0 / 0	1 / 0	0 / 0	2 / 0	2 / 0	6 / 0	4 / 0	2 / 0	5 / 1	2 / 1	6 / 1	2 / 1	3 / 0	0 / 1
40	0 / 0	0 / 0	0 / 0	0 / 0	0 / 0	0 / 0	1 / 0	0 / 0	0 / 0	0 / 0	1 / 0	1 / 0	2 / 0	2 / 0	3 / 0	7 / 0	4 / 1	4 / 0	1 / 0	0 / 0	1 / 0

	21	22	23	24	25	26	27	28	29	30	31	32	33	34	35	36	37	38	39	40
5																				
6																				
7																				
8																				
9																				
10																				
11																				
12																				
13																				
14																				
15																				
16																				
17																				
18																				
19																				
20																				
21	4 / 2																			
22	1 / 0	2 / 0																		
23	0 / 0	1 / 1	3 / 0																	
24	0 / 0	0 / 0	1 / 0	2 / 1																
25	0 / 0	0 / 0	0 / 0	1 / 0	2 / 0															
26	0 / 0	0 / 0	0 / 0	0 / 0	0 / 0	1 / 0														
27	0 / 1	0 / 0	0 / 0	0 / 0	0 / 0	1 / 1	2 / 0													
28	0 / 0	0 / 0	1 / 0	0 / 0	0 / 0	1 / 0	1 / 0	0 / 0												
29	0 / 0	0 / 0	0 / 0	0 / 0	0 / 0	0 / 0	1 / 0	0 / 0	1 / 2											
30	0 / 0	0 / 0	0 / 0	0 / 0	0 / 0	0 / 0	0 / 0	0 / 0	1 / 0	1 / 0										
31	0 / 0	0 / 0	0 / 0	0 / 0	0 / 0	2 / 0	0 / 0	0 / 0	0 / 0	0 / 0	0 / 1									
32	0 / 0	1 / 0	0 / 0	0 / 0	0 / 0	0 / 0	0 / 0	0 / 0	0 / 0	0 / 0	0 / 0	1 / 0								
33	0 / 0	0 / 0	0 / 0	0 / 0	0 / 0	0 / 0	0 / 0	0 / 0	0 / 0	0 / 0	0 / 0	0 / 0	1 / 0							
34	0 / 0	0 / 0	0 / 0	0 / 0	0 / 0	0 / 0	0 / 0	0 / 0	0 / 0	0 / 0	0 / 0	0 / 0	0 / 0	0 / 0						
35	0 / 0	0 / 0	0 / 0	0 / 0	0 / 0	0 / 0	0 / 0	0 / 0	1 / 0	0 / 0	0 / 0	0 / 0	0 / 0	0 / 0	0 / 0					
36	0 / 0	1 / 0	0 / 0	0 / 0	1 / 0	0 / 0	0 / 0	0 / 0	0 / 0	0 / 0	0 / 0	0 / 0	0 / 0	0 / 0	0 / 0	0 / 0				
37	1 / 0	1 / 0	1 / 0	0 / 0	0 / 0	0 / 0	0 / 0	0 / 0	0 / 0	0 / 0	0 / 0	0 / 0	0 / 0	0 / 0	0 / 0	0 / 0	0 / 0			
38	0 / 0	0 / 0	0 / 0	0 / 0	0 / 0	0 / 0	0 / 0	0 / 0	0 / 0	0 / 0	0 / 0	0 / 0	0 / 0	0 / 0	0 / 0	0 / 0	0 / 0	0 / 0		
39	0 / 0	0 / 0	0 / 0	0 / 0	0 / 0	0 / 0	0 / 0	0 / 0	0 / 0	0 / 0	0 / 0	0 / 0	0 / 0	0 / 0	0 / 0	0 / 0	0 / 0	0 / 0	0 / 0	
40	1 / 0	3 / 0	1 / 0	0 / 0	0 / 0	0 / 0	0 / 0	0 / 0	0 / 0	0 / 0	0 / 0	0 / 0	0 / 0	0 / 0	0 / 0	0 / 0	0 / 0	0 / 0	0 / 0	0 / 0

Table S2. Microinversions detected in the human-chimpanzee genome alignment. Rows shows the length of a "bubble" from 5 to 40 nucleotides; columns show the number of matches between the reverse complement of chimpanzee DNA sequence and the corresponding human DNA sequence. Microinversions that we consider to be true are highlighted yellow, probable microinversions are highlighted grey. In each cell, the number of "bubbles" found in the original alignment is shown before dash; the number of "bubbles" found in realigned segments (which were not presented in the original alignment) is after dash.

Number of matches between the reverse complement of bonobo DNA sequence and the corresponding chimpanzee DNA sequence

Length of a "bubble"

	0	1	2	3	4	5	6	7	8	9	10	11	12	13	14	15	16	17	18	19	20	21	22	23	24	25	26	27	28	29	30	31	32	33	34	35	36	37	38	39	40
5	0 / 7	0 / 6	0 / 10	0 / 5	0 / 2	0 / 0																																			
6	4 / 3	2 / 13	3 / 9	5 / 4	0 / 0	0 / 0	0 / 0																																		
7	3 / 1	6 / 7	8 / 12	13 / 10	5 / 3	1 / 0	0 / 0	0 / 0																																	
8	307 / 4	576 / 4	811 / 8	642 / 4	338 / 4	115 / 1	33 / 0	6 / 0	4 / 0																																
9	139 / 2	327 / 6	550 / 10	491 / 7	360 / 5	161 / 3	43 / 0	7 / 0	1 / 0	1 / 0																															
10	160 / 0	151 / 3	338 / 7	383 / 6	388 / 4	233 / 2	74 / 0	19 / 0	2 / 0	0 / 0	0 / 0																														
11	51 / 0	98 / 0	215 / 2	286 / 3	363 / 7	201 / 5	78 / 2	16 / 0	6 / 0	0 / 0	0 / 0	1 / 0																													
12	24 / 0	39 / 0	138 / 5	197 / 3	276 / 6	170 / 4	115 / 3	35 / 0	14 / 0	3 / 0	0 / 1	0 / 0	0 / 2																												
13	15 / 0	22 / 1	76 / 2	143 / 6	169 / 3	188 / 7	120 / 5	43 / 1	15 / 0	4 / 0	3 / 0	0 / 0	0 / 0	1 / 0																											
14	17 / 0	18 / 1	42 / 0	94 / 5	138 / 4	139 / 2	115 / 4	67 / 2	26 / 1	6 / 0	3 / 0	0 / 0	0 / 0	0 / 0	0 / 1																										
15	11 / 0	13 / 2	23 / 1	63 / 4	89 / 0	96 / 2	114 / 1	58 / 2	32 / 1	10 / 1	1 / 0	2 / 0	0 / 0	0 / 0	0 / 0	0 / 0																									
16	10 / 0	7 / 0	13 / 0	28 / 0	66 / 1	85 / 4	84 / 6	50 / 1	31 / 1	19 / 1	11 / 0	3 / 0	1 / 0	0 / 0	0 / 0	0 / 0	0 / 0																								
17	15 / 0	7 / 0	14 / 1	25 / 0	35 / 1	45 / 1	62 / 1	52 / 5	37 / 2	9 / 2	4 / 0	2 / 0	0 / 0	0 / 0	0 / 0	0 / 0	0 / 0	1 / 0																							
18	16 / 0	8 / 0	12 / 0	14 / 0	27 / 0	40 / 1	54 / 2	55 / 0	40 / 2	20 / 1	9 / 0	1 / 0	0 / 0	1 / 1	0 / 0	0 / 0	0 / 0	0 / 0	0 / 0																						
19	7 / 0	7 / 0	3 / 0	7 / 0	17 / 1	35 / 0	31 / 1	39 / 2	37 / 1	23 / 2	12 / 2	8 / 0	1 / 0	0 / 0	0 / 0	0 / 0	0 / 0	0 / 0	0 / 0	0 / 0																					
20	111 / 0	3 / 0	5 / 0	6 / 0	16 / 0	12 / 1	40 / 2	39 / 1	30 / 3	19 / 0	16 / 0	5 / 0	2 / 0	1 / 0	1 / 0	0 / 0	0 / 0	0 / 0	0 / 0	0 / 0	0 / 0																				
21	17 / 0	7 / 0	3 / 0	8 / 0	9 / 0	17 / 1	22 / 1	23 / 2	31 / 2	18 / 0	22 / 1	8 / 1	5 / 1	0 / 0	1 / 0	0 / 0	0 / 0	0 / 0	0 / 0	0 / 1	0 / 0	0 / 0																			
22	12 / 0	7 / 0	4 / 0	1 / 0	6 / 1	7 / 0	14 / 1	18 / 1	26 / 0	20 / 1	14 / 0	6 / 2	4 / 1	2 / 0	2 / 0	0 / 0	1 / 0	0 / 0	0 / 0	0 / 0	1 / 0	0 / 0	0 / 0																		
23	6 / 0	2 / 0	2 / 0	2 / 0	1 / 0	5 / 0	7 / 1	17 / 0	23 / 0	20 / 0	10 / 0	8 / 1	8 / 0	1 / 0	0 / 0	0 / 0	0 / 0	0 / 0	0 / 0	0 / 0	0 / 0	0 / 0	0 / 0	1 / 0																	
24	6 / 0	4 / 0	2 / 0	1 / 0	5 / 0	2 / 0	11 / 1	15 / 0	13 / 1	13 / 0	6 / 1	7 / 0	12 / 0	1 / 0	1 / 0	1 / 0	0 / 0	0 / 0	0 / 0	0 / 1	0 / 0	0 / 0	1 / 0	0 / 0	0 / 1																
25	3 / 0	4 / 0	2 / 0	4 / 0	6 / 1	4 / 0	8 / 0	8 / 0	20 / 1	14 / 1	9 / 0	7 / 2	7 / 1	1 / 0	1 / 0	0 / 0	0 / 0	0 / 0	0 / 0	0 / 0	0 / 0	0 / 0	0 / 0	1 / 0	0 / 0	0 / 1															
26	13 / 0	1 / 0	0 / 0	2 / 0	2 / 0	6 / 0	2 / 0	5 / 0	12 / 0	10 / 2	11 / 0	6 / 2	4 / 0	3 / 0	2 / 0	1 / 0	0 / 0	0 / 0	0 / 0	0 / 0	0 / 0	0 / 0	0 / 0	0 / 0	0 / 0	0 / 0	0 / 0														
27	4 / 0	3 / 0	5 / 0	2 / 0	2 / 0	3 / 0	2 / 0	5 / 0	11 / 1	8 / 1	10 / 0	9 / 0	7 / 0	4 / 0	1 / 0	0 / 0	0 / 0	1 / 0	0 / 0	0 / 0	0 / 0	0 / 0	0 / 0	0 / 0	0 / 0	0 / 0	0 / 0	0 / 0													
28	8 / 0	0 / 0	3 / 0	0 / 0	1 / 0	1 / 0	6 / 0	0 / 0	3 / 0	5 / 0	6 / 1	4 / 0	3 / 2	6 / 0	7 / 1	4 / 0	0 / 0	0 / 0	0 / 0	0 / 0	0 / 0	0 / 0	0 / 0	0 / 0	1 / 0	0 / 0	0 / 0	0 / 0	0 / 0												
29	11 / 0	0 / 0	1 / 0	2 / 0	3 / 0	1 / 0	0 / 0	4 / 0	5 / 0	3 / 0	5 / 0	6 / 1	7 / 0	6 / 0	4 / 0	2 / 0	2 / 0	0 / 0	0 / 0	0 / 0	0 / 0	0 / 0	0 / 0	0 / 0	0 / 0	0 / 0	0 / 0	0 / 0	0 / 0	0 / 0											
30	26 / 0	1 / 0	4 / 0	1 / 0	0 / 0	3 / 0	3 / 0	2 / 0	2 / 0	3 / 0	8 / 0	3 / 0	6 / 1	6 / 1	5 / 0	1 / 0	3 / 0	0 / 1	0 / 0	0 / 0	0 / 0	0 / 0	0 / 0	0 / 0	0 / 0	0 / 0	0 / 0	0 / 0	0 / 0	0 / 0	0 / 0										
31	9 / 0	4 / 0	1 / 0	3 / 0	2 / 0	1 / 0	1 / 0	1 / 0	1 / 0	6 / 1	2 / 0	6 / 0	5 / 1	9 / 2	3 / 0	2 / 1	1 / 0	0 / 0	0 / 0	0 / 0	0 / 0	0 / 0	0 / 0	0 / 0	0 / 0	0 / 0	0 / 0	0 / 0	0 / 0	0 / 0	0 / 0	0 / 0									
32	8 / 0	2 / 0	3 / 0	2 / 0	0 / 0	1 / 0	1 / 0	2 / 0	0 / 0	4 / 0	2 / 0	7 / 0	1 / 0	6 / 0	6 / 1	4 / 0	4 / 0	0 / 1	2 / 0	0 / 0	0 / 0	0 / 0	0 / 0	0 / 0	0 / 0	0 / 0	0 / 0	0 / 0	0 / 0	0 / 0	0 / 0	0 / 0	0 / 0								
33	9 / 0	1 / 0	0 / 0	0 / 0	1 / 0	0 / 0	0 / 0	1 / 0	1 / 0	3 / 0	4 / 0	2 / 1	3 / 0	8 / 1	3 / 0	2 / 0	0 / 0	1 / 0	0 / 0	0 / 0	0 / 0	0 / 0	0 / 0	0 / 0	0 / 0	0 / 0	0 / 0	0 / 0	0 / 0	0 / 0	0 / 0	0 / 0	0 / 0	1 / 0							
34	8 / 0	2 / 0	0 / 0	1 / 0	0 / 0	1 / 0	0 / 0	0 / 0	3 / 0	1 / 0	1 / 0	3 / 0	6 / 0	2 / 0	2 / 0	4 / 0	0 / 0	2 / 0	1 / 0	0 / 0	0 / 0	0 / 0	0 / 0	0 / 0	0 / 0	0 / 0	0 / 0	0 / 0	0 / 0	0 / 0	0 / 0	0 / 0	0 / 0	0 / 0	0 / 0						
35	14 / 0	2 / 0	1 / 0	2 / 0	1 / 1	1 / 0	1 / 0	1 / 0	0 / 0	0 / 0	0 / 0	1 / 1	3 / 0	4 / 0	3 / 0	1 / 1	2 / 1	1 / 0	3 / 1	0 / 0	0 / 0	0 / 0	0 / 1	0 / 0	1 / 0	0 / 0	0 / 0	0 / 0	1 / 0	0 / 0	0 / 0	0 / 0	0 / 0	0 / 0	0 / 0	0 / 0					
36	10 / 0	4 / 0	3 / 0	0 / 0	1 / 0	0 / 0	0 / 0	2 / 0	0 / 0	0 / 0	2 / 0	2 / 0	0 / 0	0 / 0	5 / 0	2 / 1	1 / 0	2 / 0	1 / 0	1 / 0	1 / 0	0 / 0	0 / 0	0 / 0	0 / 0	0 / 0	0 / 0	0 / 0	0 / 0	0 / 0	0 / 0	0 / 0	0 / 0	0 / 0	0 / 0	0 / 0	0 / 0				
37	10 / 0	2 / 0	3 / 0	2 / 0	0 / 0	1 / 0	1 / 0	0 / 0	0 / 0	0 / 0	1 / 0	2 / 0	3 / 0	3 / 0	1 / 0	3 / 0	1 / 0	1 / 0	0 / 0	1 / 0	0 / 0	0 / 0	0 / 0	0 / 0	0 / 0	0 / 0	0 / 0	0 / 0	0 / 0	0 / 0	0 / 0	0 / 0	0 / 0	0 / 0	0 / 0	0 / 0	0 / 0	0 / 0			
38	6 / 0	2 / 0	1 / 0	2 / 0	0 / 0	0 / 0	1 / 0	1 / 0	1 / 0	2 / 0	1 / 0	2 / 0	2 / 0	0 / 0	4 / 0	3 / 0	2 / 1	1 / 0	0 / 1	1 / 0	0 / 0	0 / 0	0 / 0	0 / 0	0 / 0	0 / 0	0 / 0	0 / 0	0 / 0	0 / 0	0 / 0	0 / 0	0 / 0	0 / 0	0 / 0	0 / 0	0 / 0	0 / 0	0 / 0		
39	4 / 0	1 / 0	4 / 0	2 / 0	0 / 0	0 / 0	0 / 0	0 / 0	1 / 0	0 / 0	0 / 0	2 / 0	0 / 0	0 / 0	1 / 0	2 / 0	3 / 0	1 / 0	2 / 0	1 / 0	0 / 0	0 / 0	0 / 1	0 / 0	0 / 0	0 / 0	0 / 0	0 / 0	0 / 0	0 / 0	0 / 0	0 / 0	0 / 0	0 / 0	0 / 0	0 / 0	0 / 0	0 / 0	0 / 0	0 / 0	
40	4 / 0	0 / 0	0 / 0	0 / 0	0 / 0	1 / 0	0 / 0	1 / 0	0 / 0	0 / 0	1 / 0	1 / 0	0 / 0	2 / 0	1 / 0	2 / 0	1 / 1	0 / 0	1 / 0	0 / 0	0 / 0	0 / 0	1 / 0	0 / 0	0 / 0	0 / 0	0 / 0	0 / 0	0 / 0	0 / 0	0 / 0	0 / 0	0 / 0	0 / 0	0 / 0	0 / 0	0 / 0	0 / 0	0 / 0	0 / 0	0 / 0

Table S3. Microinversions in the chimpanzee-bonobo genome alignment. Rows correspond to the length of a "bubble" from 5 to 40 nucleotides; columns correspond to the number of matches between the reverse complement of bonobo DNA sequence and the corresponding chimpanzee DNA sequence. Microinversions that we consider to be true are highlighted yellow, probable microinversions are highlighted grey. In each cell, the number of "bubbles" found in the original alignment is shown before dash; the number of "bubbles" found in realigned segments (which were not presented in the original alignment) is after dash.
